# The Inflammasome Signaling Proteins ASC and IL-18 as Biomarkers of Psoriasis

**DOI:** 10.3389/fphar.2020.01238

**Published:** 2020-08-11

**Authors:** Mahtab Forouzandeh, Jaren Besen, Robert W. Keane, Juan Pablo de Rivero Vaccari

**Affiliations:** ^1^ The Department of Dermatology and Cutaneous Surgery, University of Miami Miller School of Medicine, Miami, FL, United States; ^2^ Department of Neurological Surgery and The Miami Project to Cure Paralysis, University of Miami Miller School of Medicine, Miami, FL, United States; ^3^ Department of Physiology and Biophysics, University of Miami Miller School of Medicine, Miami, FL, United States

**Keywords:** psoriasis, biomarkers, inflammasome, inflammation, caspase-1, interleukin-18, ASC

## Abstract

Inflammasome activation in the innate immune response plays a role in the pathogenesis of psoriasis largely due to the increased levels of pro-inflammatory cytokines. However, the precise role of inflammasomes in psoriasis (Ps) and psoriatic arthritis (PsA) is largely undefined. To establish the reliability of inflammasome signaling proteins as diagnostics and predictive biomarkers of clinical severity in this disease population, serum from healthy donors and patients with Ps/PsA were analyzed for the protein expression of caspase-1, apoptosis-associated speck-like protein containing a caspase-recruitment domain (ASC), interleukin (IL)-1β and IL-18 levels to determine cut-off points, positive and negative predictive values, and receiver operator characteristic (ROC) curves. Our data revealed that ASC and IL-18 proteins were significantly higher in the Ps group when compared to healthy controls. The area under the curve (AUC) for ASC was 0.9224 with a cut-off point of 321.8 pg/ml, while IL-18 had an AUC of 0.7818 and a cut-off point of 232.1 pg/ml. In addition, levels of IL-18 had a statistically significant linear correlation with that of ASC with an adjusted R squared of 0.2566, indicating that approximately 25% of IL-18 levels could be explained by ASC levels in serum. Our findings indicate that ASC and IL-18 play a significant role in the inflammatory response associated with the pathology of Ps. These inflammasome proteins appear to be key biomarkers in determining diagnoses in this patient population.

## Introduction

Psoriasis (Ps) is a chronic immune-mediated systemic disease that affects over 125 million people globally and has damaging effects that extend well beyond the dermis. Ps is characterized by relapsing skin lesions, demonstrating epidermal hyperplasia, inflammatory infiltration, and angiogenesis. There is a strong relationship between Ps and a number of serious comorbidities including cardiovascular disease, metabolic syndrome, atherosclerosis, non-alcoholic fatty liver disease, lymphomas, chronic obstructive pulmonary disease, osteoporosis, Parkinson’s disease, and Celiac disease (Oliveira Mde et al., 2015). Approximately 25 to 30% of patients with Ps also suffer from psoriatic arthritis (PsA). PsA is a type of inflammatory arthritis that typically coexists with the cutaneous findings of Ps, usually manifesting as a mono or asymmetrical oligo-arthritis in the absence of the rheumatoid factor (Alinaghi et al., 2019).

A genetic component associated with the inflammasome has been previously described in psoriasis susceptibility (Carlstrom et al., 2012). In addition, in animals models of psoriasis, the inflammasome has also been described as a key modulator of the inflammatory response (Hu et al., 2013; Jiang et al., 2013; Goblos et al., 2016). These findings suggest that Ps pathogenesis involves the activation of the inflammasome multiprotein complex, which is involved in the production of interleukin (IL)-1β and IL-18, two inflammatory cytokines seen in Ps pathogenesis. Assembly of the inflammasome components involves inflammasome receptor interaction with the adaptor protein ASC (apoptosis-associated speck-like protein containing a caspase-recruitment domain), which then recruits pro-caspase-1 and results in the activation of the effector caspase through proteolytic cleavage. The activated caspase-1 then cleaves pro-IL-1β and pro-IL-18 to produce active forms of these pro-inflammatory cytokines (De Rivero Vaccari et al., 2014). Interestingly, recent literature reveals that polymorphisms of the NLRP1 inflammasome complex are also associated with an increased susceptibility to Ps (Ekman et al., 2014).

We have previously shown that inflammasome signaling proteins are promising biomarkers of active inflammation in other chronic diseases and systemic injuries including stroke (Kerr et al., 2018a), traumatic brain injury (Adamczak et al., 2012; Kerr et al., 2018b; Perez-Barcena et al., 2020), multiple sclerosis (Keane et al., 2018), depression (Syed et al., 2018), mild cognitive impairment (Scott et al., 2020), and Alzheimer’s disease (Scott et al., 2020). In this study, the role of the inflammasome in Ps pathogenesis was investigated through the identification of inflammasome protein levels in human serum. Specifically, we evaluated the potential for inflammasome signaling proteins to serve as biomarkers that could be used in the clinical setting to determine the diagnosis of Ps. Serum samples from healthy donors were analyzed for protein expression levels of caspase-1, ASC, IL-1β, and IL-18 and were compared to serum levels in patients with Ps. Cut-off points, positive and negative predictive values, and receiver operator characteristic (ROC) curves with associated sensitivity and specificity calculations were determined for each of these inflammasome proteins.

## Materials and Methods

### Participants

Samples for this study were purchased from BioIVT (Hicksville, NY). Informed consent was obtained from donors enrolled in the study Prospective Collection of Samples for Research sponsored by SeraTrials, LLC. with the IRB number 20170439. The age range of donors was from 21 to 79 years old with 180 samples in the control group and 37 samples in the Ps group (**Table 1**). The control group consisted of healthy age-matched individuals without any diagnosed disease.

**Table 1:** Patients used in the study.

Age	Diagnosis	Medications
60-65	Plaque Psoriasis, Hypothyroidism, Depression, Hypertension (HTN)	Synthroid 100 mcg, Atenolol 50 mg, Amitriptyline 10 mg
40-45	Plaque Psoriasis, Hypertension (HTN)	Lisinopril 20 mg
25-30	Plaque Psoriasis, Hypothyroidism, Schizophrenia	Levothyroxine 173 mcg, Atomoxetine 40 mg, Rexulti 3 mg, Enstilar 0.005%, Hydrocortisone 2.5%, Tacrolimus 0.1%
40-45	Plaque Psoriasis, Rheumatoid Arthritis (RA)	Humira 40 mg/0.8 ml, Taclonex 0.005%
55-60	Plaque Psoriasis, Hypertension (HTN)	Lisinopril 10 mg, Clobetasol 0.05%, Fluocinonide 0.05%, Hydrocortisone 2.5%
40-45	Plaque Psoriasis	Humira 40 mg, Clobetasol
55-60	Plaque Psoriasis	Cortizone
60-65	Plaque Psoriasis, Hypertriglyceridemia, Gastroesophageal Reflux Disease (GERD), Alzheimer’s Disease (AD), Cardiovascular Disease	Diovan 160 mg, Aricept 10 mg, Vascepa 2 g, Nexium 20 mg
70-75	Plaque Psoriasis, Hypertension (HTN), Hyperchlolesterolemia, Rheumatoid Arthritis (RA), Gastroeosphageal Reflux Disease (GERD)	Atacand 32 mg, Toprol 100 mg, Nexium 40 mg, Pravastatin 20 mg, Vascepa 1 mg, Synthroid 75 mcg, Tramadol 50 mg
40-45	Plaque Psoriasis	None
40-45	Psoriasis	Coconut Oil
30-35	Plaque Psoriasis	Clobetasol
60-65	Psoriasis, Psoriatic Arthritis, Osteoarthritis (OA), Saphenofemoral Venous Reflux, Eczema	Humira 40 mg/0.8 ml, Aspirin 81 mg, Lipitor 20 mg, Vitamin D, Clobetasol 0.05%, Flexeril 5 mg, Norco 5–325mg, Multivitamin with Minerals, Vitamin B1, Kenalog 0.1%
45-50	Plaque Psoriasis, Depression	Humira 40 mg, Methotrexate 15 mg, Folic Acid 1 mg, Zoloft 50 mg, Vitamin D 1000iu, Multivitamin
35-40	Plaque Psoriasis, Gastroesophageal Reflux Disease (GERD), Anxiety, Hypercholesterolemia	Calcipotriene 0.005%, Citalopram 20 mg, Testosterone 200 mg, Pantoprazole 40 mg
65-70	Plaque Psoriasis, Allergy (Seasonal), Generalized Anxiety Disorder (GAD), Osteoarthritis (OA), Osteoporosis, Hypertension (HTN), Hypercholesterolemia	Triamcinolone 0.1%, Allegra 180 mg, Alprazolam 0.25 mg, Carvedilol 6.25 mg, Citalopram 20 mg, Diltiazem 360 mg, Hydrochlorothiazide 25 mg, Lovastatin 20 mg, Losartan 100 mg, Norco 10–325mg
60-65	Plaque Psoriasis, Gastroesophageal Reflux Disease (GERD), Depression, Osteoarthritis (OA), Allergy (Seasonal)	Bupropion 300 mg, Omeprazole 20 mg, Clobetasol 0.05%
50-55	Plaque Psoriasis	Triamcinolone 0.5%
35-40	Psoriasis	None
50-55	Psoriasis	None
75-80	Psoriasis, Hypertension (HTN), Gastroesophageal Reflux Disease (GERD), Allergy (Seasonal), Coronary Arteriosclerosis	Aspirin 81 mg, Baclofen 10 mg, Clopidogrel 75 mg, Valsartan 80 mg, CoQ-10
50-55	Plaque Psoriasis, Atrial fibrillation (AF)	Taltz 80 mg
35-40	Plaque Psoriasis, Asthma, Type 2 Diabetes, Hypothyroidism, Depression	None
50-55	Plaque Psoriasis	None
25-30	Psoriasis	Nexplanon 68 mg
70-75	Erythrodermic Psoriasis, Asthma, Type 2 Diabetes, Hypertension (HTN), Myocardial Infarction (MI), Gastroesophageal Reflux Disease (GERD), Obstructive Sleep Apnea (OSA), Hiatal Hernia	Glipizide 10 mg, Lantus 61 iu, Diltiazem 120 mg, Metoprolol 50 mg, Isosorbide Mononitrate 20 mg, Triamcinolone
65-70	Psoriasis	None
45-50	Plaque Psoriasis, Hypertension (HTN), Fibromyalgia Syndrome (FMS), Generalized Anxiety Disorder (GAD), Major Depressive Disorder (MDD)	Amitriptyline 25 mg, Buprenorphine 8 mg, Duloxetine 60 mg, Fluticasone 50 mcg, Gabapentin 600 mg, Lisinopril 20 mg, Tizanidine 4 mg, Albuterol 180 mcg
65-70	Plaque Psoriasis, Type 1 Diabetes, Hypercholesterolemia, Hypertension (HTN), Rheumatoid Arthritis (RA), Hyperthyroidism, Coronary Artery Disease (CAD)	Aspirin 81 mg, Atorvastatin 80 mg, Carvedilol 12.5 mg, Glimepiride 2 mg, Lisinopril 20 mg, Pioglitazone 45 mg, Synthroid 125 mcg, Humira 40 mg
40-45	Erythematous Plaque Psoriasis, Adenocarcinoma of Endometrium, Herpes Simplex Virus (HSV), Metabolic Syndrome X, Eczema, Anemia, Mild Non Proliferative Diabetic Retinopathy, Senile Hyperkeratosis, Rosacea, Obesity, Anxiety, Candidiasis, Hypertension (HTN), Obstructive Sleep Apnea (OSA), Neoplasm of Skin	Clobetasol 0.05%, Desonide 0.05%, Fluocinonide 0.05%, Intrarosa 6.5 mg, Metformin ER 1,000 mg, Telmisartan 80 mg-Hydrochlorothiazide 12.5 mg, Triamcinolone Acetonide 0.1%, Women’s 1-A-Day
55-60	Dialysate, Hypercalcemia, Hypocalcemia, Hyperparathyroidism, Vitamin D Deficiency, Asthma, Chronic Obstructive Pulmonary Disease (COPD), Crohn’s Disease, Ulcerative Colitis (UC), Gastroesophageal Reflux Disease (GERD), Chronic Kidney Disease (CKD), Rheumatoid Arthritis (RA), Type 1 Diabetes, Type 2 Diabetes, Lupus, Multiple Sclerosis, Bacteremia, Iron Deficient Anemia, Anemia (CKD), Hypertension (HTN), Psoriasis, Melanoma, Vitamin B Deficiency, Hyperkalemia, Obesity	Daily-Vite, Protonix 40 mg, Renvela, Tums 2,000 mg, Viagra 50 mg
20-25	Plaque Psoriasis	Triamcinolone 0.1%, Taltz 160 mg
60-65	Plaque Psoriasis, Asthma, Hypertension (HTN), Hypercholesterolemia, Insomnia, Major Depressive Disorder (MDD), Generalized Anxiety Disorder (GAD), Irritable Bowel Syndrome (IBS), Gastroesophageal Reflux Disease (GERD), Allergy (Seasonal), Osteoarthritis (OA)	Advair 250/50 mcg, Alprazolam 0.5 mg, Amitriptyline 10 mg, Cetirizine 10 mg, Lisinopril 20 mg, Meloxicam 15 mg, Montelukast 10 mg, Pantoprazole 40 mg, Pravastatin 40 mg, Progesterone 100 mg, Venlafaxine 150 mg, Albuterol 90 mcg, Vitamin D3 2,000 iu, Wellbutrin 150 mg, Hydrocortisone 2.5%, Sucralfate 1 g
60-65	Psoriasis, Irritable Bowel Syndrome (IBS), Gastroesophageal Reflux Disease (GERD), Anxiety, Osteoarthritis (OA), Restless Leg Sydrome (RLS), Allergy (Seasonal)	Triamcinolone 0.5%, Flonase 50 mg, Mirapex 0.125 mg, Viberzi 75 mg, Zantac 150 mg, Zanaflex 4 mg
60-65	Psoriasis, Type 2 Diabetes, Hypertension (HTN), Hypercholesterolemia	Atorvastatin 40 mg, Metoprolol 50 mg, Glimepiride 4 mg, Lantus 40 iu, Novolog 90 iu
40-45	Plaque Psoriasis, Gastroesophageal Reflux Disease (GERD)	Hydrocortisone 2.5%, Tums
75-80	Chronic Plaque Psoriasis, Chronic Kidney Disease, Hypertension, Psoriasis, Depression	Amlodipine 10 mg, Hydralazine 50 mg, Furosemide 10 mg, Metoprolol 25 mg, Lisinopril 10 mg, Atorvastatin 20 mg, Novolog 70/30 mg

### Simple Plex Assay

Concentrations of caspase-1, ASC, IL-1β, and IL-18 in serum samples from the Ps group and age-matched controls were analyzed using the Ella System (Protein System) (Brand et al., 2016). In short, 50 μl of diluted serum sample were loaded to each well of the cartridge, and 1 ml of washing buffer was loaded into specified wells. The assay was automatically run in triplicates and analyzed with the Simple Plex Runner Software (Protein Simple).

### Biomarker Analyses

Data obtained from the Simple Plex assay was analyzed using Prism 8 software (GraphPad). Initially, outliers were removed, followed by the calculation of column statistics and the area under curve, which provided the specificity, sensitivity, and likelihood ratio, as well as the 95% confidence interval, standard deviation, and p-value of significance. A cut-off point was identified for the different ranges of specificities and sensitivities. Positive and negative predictive values were then calculated along with accuracy.

### Statistical Analyses

Normality was tested using the D’Agostino & Pearson omnibus and Shapiro-Wilk normality tests. Differences between groups were determined either using the Mann-Whitney test when data were not normally distributed and a two-tailed t-test when data were normally distributed. The p-value of significance used was <0.05.

### Linear Regression

Linear regression analysis between ASC and IL-18 was run using RStudio/RMarkdown with the following libraries: MASS, dplyr, ggplot, car, and broom. Data sets were transformed using a logarithmic transformation. An adjusted r-squared value was obtained to explain the approximate contribution of ASC to IL-18 protein levels. Models were evaluated using residual analysis.

### Logistic Regression

A binomial logistic regression analyses of the probability of a patient having Ps/PsA (separated into biologic-treated, non-biologic treated group, and untreated) as determined by the levels of ASC and IL-18 were run using RStudio/RMarkdown software. Models were evaluated by comparing the Akaike information criterion (AIC) value among the different tested models.

## Results

### ASC and IL-18 Are Elevated in the Serum of Patients With Psoriasis and Psoriatic Arthritis

**Figure 1:**
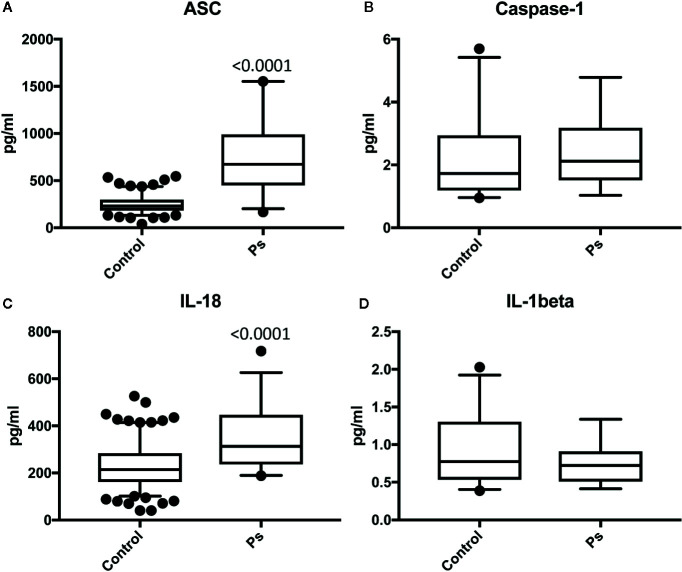
ASC and IL-18 are elevated in the serum of patients with psoriasis. Protein levels in pg/ml of ASC **(A)**, caspase-1 **(B)**, IL-18 **(C)**, and IL-1b **(D)** in serum samples from patients with psoriasis/PA and healthy donors (controls). ASC: N = 156 controls, 36 psoriasis/PA; caspase-1: N = 25 controls, 19 psoriasis/PA; IL-18: N = 180 controls, 36 psoriasis/PA IL-1beta: N = 32 controls, 14 psoriasis/PA. Box and whiskers are shown for the 5^th^ and 95^th^ percentile.

Serum samples from patients with Ps and aged-matched healthy donors were analyzed for the protein expression levels of ASC (**Figure 1A**), caspase-1 (**Figure 1B**), IL-18 (**Figure 1C**), and IL-1β (**Figure 1D**). ASC and IL-18 proteins were significantly higher in the Ps group when compared to controls. These findings indicate that ASC and IL-18 play a significant role in the inflammatory response in the pathology of Ps.

### ASC as a Prominent Biomarker of Psoriasis and Psoriatic Arthritis

**Figure 2:**
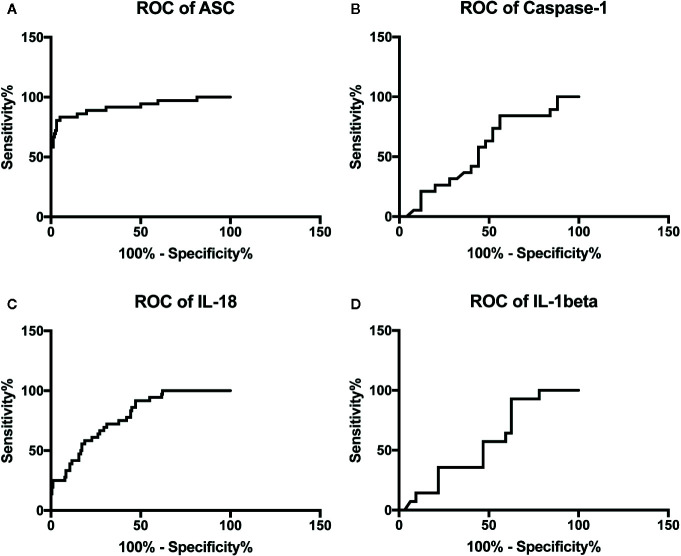
ROC curves for caspase-1 **(A)**, ASC **(B)**, IL-1b **(C)**, and IL-18 **(D)** from serum samples of patients with psoriasis/PA and healthy donors.

To determine if inflammasome signaling proteins were reliable biomarkers of active disease in Ps, the area under the curve (AUC) was calculated for caspase-1 (**Figure 2A**), ASC (**Figure 2B**), IL-1β (**Figure 2C**), and IL-18 (**Figure 2D**). Of the proteins that were analyzed, ASC had the highest AUC of 0.9224 (p < 0.0001). IL-18 had an AUC of 0.7818 (p < 0.0001) (**Table 2**). Moreover, ASC had a cut-off point of 321.8 pg/ml with 89% sensitivity and 80% specificity (**Table 3**). Comparatively, the cut-off point for IL-18 was 232.1 pg/ml with a sensitivity of 78% and a specificity of 58% (**Table 3**). These findings indicate that ASC and IL-18 have the characteristics of reliable biomarkers of the inflammatory response associated with Ps.

**Table 2:** ROC summary.

Biomarker	Area	Std. error	95% C.I.	P value
**ASC**	0.9224	0.03128	0.8611 to 0.9837	<0.0001
**Caspase-1**	0.5684	0.08756	0.3968 to 0.7400	0.4413
**IL-18**	0.7818	0.03721	0.7089 to 0.8547	<0.0001
**IL-1beta**	0.5658	0.08684	0.3957 to 0.7360	0.4813

**Table 3:** Biomarker characteristics.

Biomarker	Cut-off point (pg/ml)	Sensitivity(%)	Specificity(%)	LR	PPV(%)	NPV(%)	Accuracy(%)
**ASC**	>321.8	89	80	4.473	51	97	82
**Caspase-1**	>1.569	74	48	1.417	52	71	59
**IL-18**	>232.1	78	58	1.842	27	93	61
**IL-1beta**	<0.88	64	41	1.083	32	72	48

### Linear Regression Between ASC and IL-18

**Figure 3:**
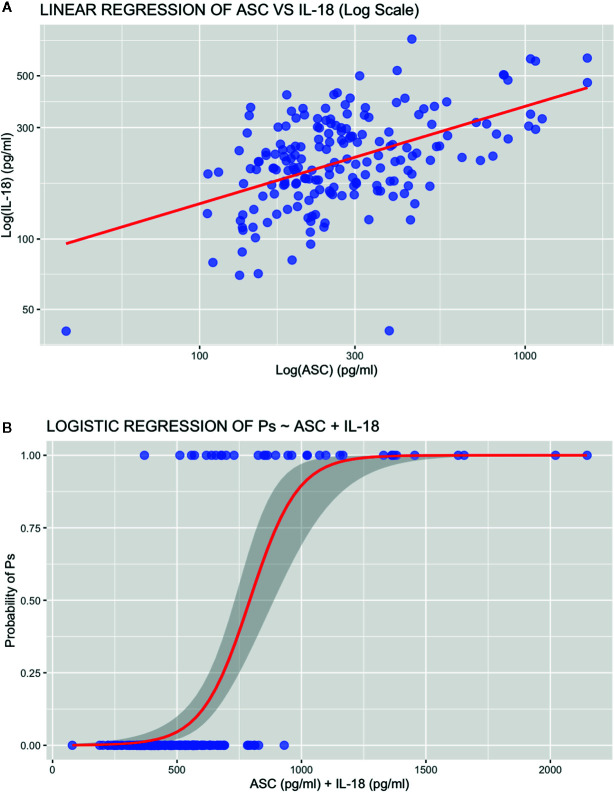
Regression analyses. **(A)** Linear regression plot of ASC vs IL-18. **(B)** Logistic regression plot of psoriasis ~ ASC + IL-18.

A linear regression analysis was run to determine the relationship between ASC and IL-18. A linear model was fit to the plotted data (**Figure 3A**). Levels of IL-18 had a statistically significant linear correlation with that of ASC (5.36 e-14) with an adjusted R squared of 0.2566 (**Supplementary Figure 1**). A logarithmic transformation was used to normalized the distribution of the data. Further fitting of the model was evaluated by analyzing the residuals (**Supplementary Figure 2**). The results indicate that 25% of the levels of IL-18 could be explained by ASC. Thus, the data show that approximately a quarter of IL-18 present in serum can be explained by ASC protein levels in serum, with the remainder being due to other proteins that were not included in this statistical model.

### Logistic Regression Between Psoriasis and ASC and IL-18

To predict the probability that protein levels of ASC and IL-18 contribute to the pathology of Ps, we run a binomial logistic regression for the proteins levels of ASC and IL-18 in serum of patients with and without a Ps diagnosis (**Figure 3B**). Accordingly, the odds of having Ps increased with increased protein levels of ASC and IL-18 as determined by an estimated coefficient of 0.012721 (p = 3.04 e-7) and 0.005947 (p = 0.0421 (**Supplementary Figure 3**), respectively. Thus, indicating that, as protein levels of ASC and IL-18 increase, so do the odds of a patient having psoriasis.

## Discussion

**Psoriasis **is an immune-mediated inflammatory disease that involves a complex network of cytokines and chemokines produced by various types of immune cells. Inflammasomes are multiprotein cytoplasmic complexes with a fundamental role in the innate immune response (De Rivero Vaccari et al., 2014). They consist of a sensor protein such as NOD-like receptor (NLRP1, NLPR3), an adaptor protein (ASC) and an effector protein (caspases-1, -5, -11) (Martinon et al., 2002). Inflammasome assembly leads to an inflammatory response resulting in the production and release of IL-1β and IL-18 (Martinon et al., 2002). To date, the role of the inflammasome and its associated inflammatory proteins in Ps and PsA remains largely undefined. Moreover, most biomarkers that have been studied in Ps/PsA do not meet the criteria for reliable biomarkers, therefore lacking clinical utility. Thus, here, we determined the expression levels of inflammasome components in patient serum samples and evaluated the reliability of the inflammasome signaling proteins ASC, caspase-1, IL-18, and IL-1β to serve as clinically useful disease biomarkers of Ps.

Our results indicate that ASC and IL-18 protein levels were significantly higher in patients with Ps when compared to healthy controls. Levels of IL-18 had a statistically significant linear correlation with that of ASC and it was determined that 25% of the IL-18 levels could be explained by ASC levels. IL-18, a potent pro-inflammatory cytokine, has been shown to promote the development and maintenance of Th17 cells, which are widely implicated in autoimmune inflammatory diseases like Ps and PsA (Sedimbi et al., 2013). An upregulation of IL-18 has also been previously demonstrated in psoriatic lesions, correlating significantly with disease duration and clinical severity (Debets et al., 1995; Rasmy et al., 2011). Thus, the elevated serum IL-18 levels measured in Ps subjects indicate that this inflammatory cytokine plays an important role in disease pathology. In addition, pronounced ASC mRNA expression has been previously demonstrated in non-lesional as well as lesional psoriatic epidermis (Salskov-Iversen et al., 2011); therefore, the substantial upregulation seen in our study among Ps serum samples supports the idea that ASC may serve as an indicator of active disease in Ps.

A genetic predisposition and several environmental triggers (e.g., physical and emotional stress, medications, infections) have been implicated in the initial stages of Ps and PsA (Guo et al., 2015). While the precise pathogenesis of Ps/PsA is not fully understood, it appears that a complex network of cytokines and chemokines produced by various types of immune cells play a major role in Ps pathology. Recent studies have focused on the identification of biomarkers in Ps to facilitate understanding of the pathogenesis, diagnosis, prognosis, and therapeutic response of the disease. Of note, identification of biomarkers related to specific Ps comorbidities, such as cardiovascular disease and the metabolic syndrome, is also of special clinical interest (Kerr et al., 2018b). It has been hypothesized that increased levels of pro-inflammatory factors seen in Ps may help explain a link to cardiovascular disease (Nickoloff, 1991). Previous studies have shown a significant overlap between the cytokines seen in Ps and those associated with atherosclerosis.

The inflammasome sensor proteins NLRP-1 and NLRP-3 are expressed in psoriatic lesions and specific polymorphisms have been associated with psoriasis pathogenesis and susceptibility (Ekman et al., 2014). However, future studies are needed to understand which inflammasomes (NLRP-1, NLRP-3, AIM-2) contribute to significant elevations in the inflammatory cytokine profile in serum of Ps individuals. Furthermore, an increase in samples size, separation between Ps and PsA samples, correlation between protein expression and clinical severity, and knowledge of disease duration and treatment duration would also render a better understanding of the role of inflammasomes in Ps.

A recent study reported that IL-18 expression levels in psoriatic skin lesions was higher in patients with active disease when compared to patients with stable disease (Companjen et al., 2004). In addition, levels of IL-18 have been previously shown to be elevated in the serum patients with psoriasis (Gangemi et al., 2003). Therefore, a similar comparison evaluating a range of inflammasome protein levels in serum samples before and after treatment could help guide therapeutic treatment strategies and aid in determination of patient prognoses. Limitations of our study include unknown clinical severity among patient samples, unknown duration of disease among samples, and unknown treatment durations. Therefore, future studies will aim to address these limitations. Moreover, future studies will look into further dividing samples into untreated and treated groups, as well as stratifying treated patients between those that were treated with biologics than those that were treated with other therapeutics.

In this study, the logistic regression model was developed for the diagnosis of Ps and patient selection was also powered for the same diagnosis and not of other comorbidities, indicating that the significant changes and values presented in this study are due to Ps. However, as stated, Ps patients tend to present with other comorbidities. Hence, it is likely that comorbidities also contribute to the levels of ASC and IL-18 detected in the serum of patients used.

Furthermore, besides analyzing protein levels, in future studies we will also analyze miRNAs that have been shown to affect inflammasome signaling (Wang et al., 2009; Pan et al., 2018; Cho et al., 2020) since miRNAs in extracellular vesicles have been recently shown to be useful biomarkers of psoriasis (Wang et al., 2020), and we have shown that inflammasome proteins in extracellular vesicles are good biomarkers of stroke (Kerr et al., 2018a). For instance, silencing of miR-155 is able to downregulate inflammasome signaling in Ps; thus, suggesting that miRNAs play an important role in Ps (Luo et al., 2018).

Taken together, our findings indicate that ASC and IL-18 play a significant role in the inflammatory response underlying the pathology of Ps. Accordingly, the AUC for ASC was 0.9224 and for IL-18 was 0.7818. Thus, these proteins appear to be reliable inflammatory biomarkers that could then be used clinically to facilitate screening and diagnosis, determine disease prognosis and systemic severity, and evaluate therapeutic response among patients with Ps. Identification of reliable biomarkers in Ps will undoubtedly provide valuable insight regarding disease susceptibility and mechanisms involved in the pathogenesis of disease progression. Such biomarkers could ultimately function as surrogate endpoints for a wide range of clinical outcomes, including optimizing patient care. Given the number of serious conditions associated with Ps (e.g., cardiovascular diseases and metabolic disorders), the identification of biomarkers that could help predict the development of Ps-related comorbidities, would greatly improve patient morbidity.

## Data Availability Statement

The raw data supporting the conclusions of this article will be made available by the authors, without undue reservation.

## Ethics Statement

The studies involving human participants were reviewed and approved by Schulman Associates IRB. The patients/participants provided their written informed consent to participate in this study.

## Author Contributions

MF, JB, and JR performed the research. MF, RK, and JR designed the research study. All authors analyzed the data and wrote the paper.

## Funding

This project was supported by funds from the Miami Project to Cure Paralysis.

## Conflict of Interest

JV and RK are co-founders and managing members of InflamaCORE, LLC, and have patents on inflammasome proteins as biomarkers of injury and disease as well as on targeting inflammasome proteins for therapeutic purposes. JV and RK are scientific advisory board members of ZyVersa Therapeutics.

The remaining authors declare that the research was conducted in the absence of any commercial or financial relationships that could be construed as a potential conflict of interest.

## Supplementary Material

Supplementary Figure 1 Linear regression model fit results for Log(IL-18) ~ Log(ASC).

Supplementary Figure 2 Residual analysis results for the model fit for Log(IL-18) ~ Log(ASC).

Supplementary Figure 3 Logistic regression model fit results for Ps ~ ASC + IL-18.

## References

[B1] Adamczak S. Dale G. De Rivero Vaccari J. P. Bullock M. R. Dietrich W. D. Keane R. W. (2012). Inflammasome proteins in cerebrospinal fluid of brain-injured patients as biomarkers of functional outcome: clinical article. J. Neurosurg. 117, 1119–1125. 10.3171/2012.9.JNS12815 23061392 PMC3576729

[B2] Alinaghi F. Calov M. Kristensen L. E. Gladman D. D. Coates L. C. Jullien D. (2019). Prevalence of psoriatic arthritis in patients with psoriasis: A systematic review and meta-analysis of observational and clinical studies. J. Am. Acad. Dermatol. 80 251-265, e219. 10.1016/j.jaad.2018.06.027 29928910

[B3] Brand F. J. 3. Forouzandeh M. Kaur H. Travascio F. De Rivero Vaccari J. P. (2016). Acidification changes affect the inflammasome in human nucleus pulposus cells. J. Inflammation (Lond) 13, 29. 10.1186/s12950-016-0137-0 PMC4997758 27563282

[B4] Carlstrom M. Ekman A. K. Petersson S. Soderkvist P. Enerback C. (2012). Genetic support for the role of the NLRP3 inflammasome in psoriasis susceptibility. Exp. Dermatol. 21, 932–937. 10.1111/exd.12049 23171454

[B5] Cho S. J. Lee M. Stout-Delgado H. W. Moon J. S. (2020). DROSHA-Dependent miRNA and AIM2 Inflammasome Activation in Idiopathic Pulmonary Fibrosis. Int. J. Mol. Sci. 21 (5), 1668. 10.3390/ijms21051668 PMC7084700 32121297

[B6] Companjen A. Van Der Wel L. Van Der Fits L. Laman J. Prens E. (2004). Elevated interleukin-18 protein expression in early active and progressive plaque-type psoriatic lesions. Eur. Cytokine Netw. 15, 210–216. 15542445

[B7] De Rivero Vaccari J. P. Dietrich W. D. Keane R. W. (2014). Activation and regulation of cellular inflammasomes: gaps in our knowledge for central nervous system injury. J. Cereb. Blood Flow Metab. 34, 369–375. 10.1038/jcbfm.2013.227 24398940 PMC3948131

[B8] Debets R. Hegmans J. P. Troost R. J. Benner R. Prens E. P. (1995). Enhanced production of biologically active interleukin-1 alpha and interleukin-1 beta by psoriatic epidermal cells ex vivo: evidence of increased cytosolic interleukin-1 beta levels and facilitated interleukin-1 release. Eur. J. Immunol. 25, 1624–1630. 10.1002/eji.1830250623 7614991

[B9] Ekman A. K. Verma D. Fredrikson M. Bivik C. Enerback C. (2014). Genetic variations of NLRP1: susceptibility in psoriasis. Br. J. Dermatol. 171, 1517–1520. 10.1111/bjd.13178 24909542

[B10] Gangemi S. Merendino R. A. Guarneri F. Minciullo P. L. Dilorenzo G. Pacor M. (2003). Serum levels of interleukin-18 and s-ICAM-1 in patients affected by psoriasis: preliminary considerations. J. Eur. Acad. Dermatol. Venereol. 17, 42–46. 10.1046/j.1468-3083.2003.00647.x 12602967

[B11] Goblos A. Danis J. Vas K. Bata-Csorgo Z. Kemeny L. Szell M. (2016). Keratinocytes express functional CARD18, a negative regulator of inflammasome activation, and its altered expression in psoriasis may contribute to disease pathogenesis. Mol. Immunol. 73, 10–18. 10.1016/j.molimm.2016.03.009 27023378

[B12] Guo H. Callaway J. B. Ting J. P. (2015). Inflammasomes: mechanism of action, role in disease, and therapeutics. Nat. Med. 21, 677–687. 10.1038/nm.3893 26121197 PMC4519035

[B13] Hu J. Yang R. Wen C. Li H. Zhao H. (2013). [Expression of NLRP3 inflammasome in BALB/c mice with imiquimod-induced psoriasis-like inflammation and therapeutic effect of mustard seed (Sinapis Alba Linn)]. Nan. Fang Yi Ke Da Xue Xue Bao 33, 1394–1398. 24067228

[B14] Jiang W. Zhu F. G. Bhagat L. Yu D. Tang J. X. Kandimalla E. R. (2013). A Toll-like receptor 7, 8, and 9 antagonist inhibits Th1 and Th17 responses and inflammasome activation in a model of IL-23-induced psoriasis. J. Invest. Dermatol. 133, 1777–1784. 10.1038/jid.2013.57 23370539

[B15] Keane R. W. Dietrich W. D. De Rivero Vaccari J. P. (2018). Inflammasome Proteins As Biomarkers of Multiple Sclerosis. Front. Neurol. 9, 135. 10.3389/fneur.2018.00135 29615953 PMC5868457

[B16] Kerr N. Garcia-Contreras M. Abbassi S. Mejias N. H. Desousa B. R. Ricordi C. (2018a). Inflammasome Proteins in Serum and Serum-Derived Extracellular Vesicles as Biomarkers of Stroke. Front. Mol. Neurosci. 11, 309. 10.3389/fnmol.2018.00309 30233311 PMC6131639

[B17] Kerr N. Lee S. W. Perez-Barcena J. Crespi C. Ibanez J. Bullock M. R. (2018b). Inflammasome proteins as biomarkers of traumatic brain injury. PloS One 13, e0210128. 10.1371/journal.pone.0210128 30596792 PMC6312377

[B18] Luo Q. Zeng J. Li W. Lin L. Zhou X. Tian X. (2018). Silencing of miR155 suppresses inflammatory responses in psoriasis through inflammasome NLRP3 regulation. Int. J. Mol. Med. 42, 1086–1095. 10.3892/ijmm.2018.3677 29767259

[B19] Martinon F. Burns K. Tschopp J. (2002). The inflammasome: a molecular platform triggering activation of inflammatory caspases and processing of proIL-beta. Mol. Cell 10, 417–426. 10.1016/S1097-2765(02)00599-3 12191486

[B20] Nickoloff B. J. (1991). The cytokine network in psoriasis. Arch. Dermatol. 127, 871–884. 10.1001/archderm.1991.01680050115015 2036036

[B21] Oliveira Mde F. Rocha Bde O. Duarte G. V. (2015). Psoriasis: classical and emerging comorbidities. Bras. Dermatol. 90, 9–20. 10.1590/abd1806-4841.20153038 PMC4323693 25672294

[B22] Pan Z. Shan Q. Gu P. Wang X. M. Tai L. W. Sun M. (2018). miRNA-23a/CXCR4 regulates neuropathic pain via directly targeting TXNIP/NLRP3 inflammasome axis. J. Neuroinflammation 15, 29. 10.1186/s12974-018-1073-0 29386025 PMC5791181

[B23] Perez-Barcena J. Crespi C. Frontera G. Llompart-Pou J. A. Salazar O. Goliney V. (2020). Levels of caspase-1 in cerebrospinal fluid of patients with traumatic brain injury: correlation with intracranial pressure and outcome. J. Neurosurg. 1–6. 10.3171/2020.2.JNS193079 32357337

[B24] Rasmy H. Mikhael N. Ismail S. (2011). Interleukin-18 expression and the response to treatment in patients with psoriasis. Arch. Med. Sci. 7, 713–719. 10.5114/aoms.2011.24144 22291810 PMC3258774

[B25] Salskov-Iversen M. L. Johansen C. Kragballe K. Iversen L. (2011). Caspase-5 expression is upregulated in lesional psoriatic skin. J. Invest. Dermatol. 131, 670–676. 10.1038/jid.2010.370 21191419

[B26] Scott X. O. Stephens M. E. Desir M. C. Dietrich W. D. Keane R. W. De Rivero Vaccari J. P. (2020). The Inflammasome Adaptor Protein ASC in Mild Cognitive Impairment and Alzheimer’s Disease. Int. J. Mol. Sci. 21 (13), 4674. 10.3390/ijms21134674 PMC7370034 32630059

[B27] Sedimbi S. K. Hagglof T. Karlsson M. C. (2013). IL-18 in inflammatory and autoimmune disease. Cell Mol. Life Sci. 70, 4795–4808. 10.1007/s00018-013-1425-y 23892891 PMC11113411

[B28] Syed S. A. Beurel E. Loewenstein D. A. Lowell J. A. Craighead W. E. Dunlop B. W. (2018). Defective Inflammatory Pathways in Never-Treated Depressed Patients Are Associated with Poor Treatment Response. Neuron 99, 914–924 e913. 10.1016/j.neuron.2018.08.001 30146307 PMC6151182

[B29] Wang W. Stassen F. R. Surcel H. M. Ohman H. Tiitinen A. Paavonen J. (2009). Analyses of polymorphisms in the inflammasome-associated NLRP3 and miRNA-146A genes in the susceptibility to and tubal pathology of Chlamydia trachomatis infection. Drugs Today (Barc) 45 (Suppl B), 95–103. 20011700

[B30] Wang Z. Y. Yan B. X. Zhou Y. Chen X. Y. Zhang J. Cai S. Q. (2020). miRNA Profiling of Extracellular Vesicles Reveals Biomarkers for Psoriasis. J. Invest. Dermatol. 10.1016/j.jid.2020.04.021 32445741

