# Supplementary figures and images for: The Inflammasome Signaling Proteins ASC and IL-18 as Biomarkers of Psoriasis
Source: Front Pharmacol. 2020 Aug 11;11:1238. doi: 10.3389/fphar.2020.01238 (PMC7438850; doi:10.3389/fphar.2020.01238)

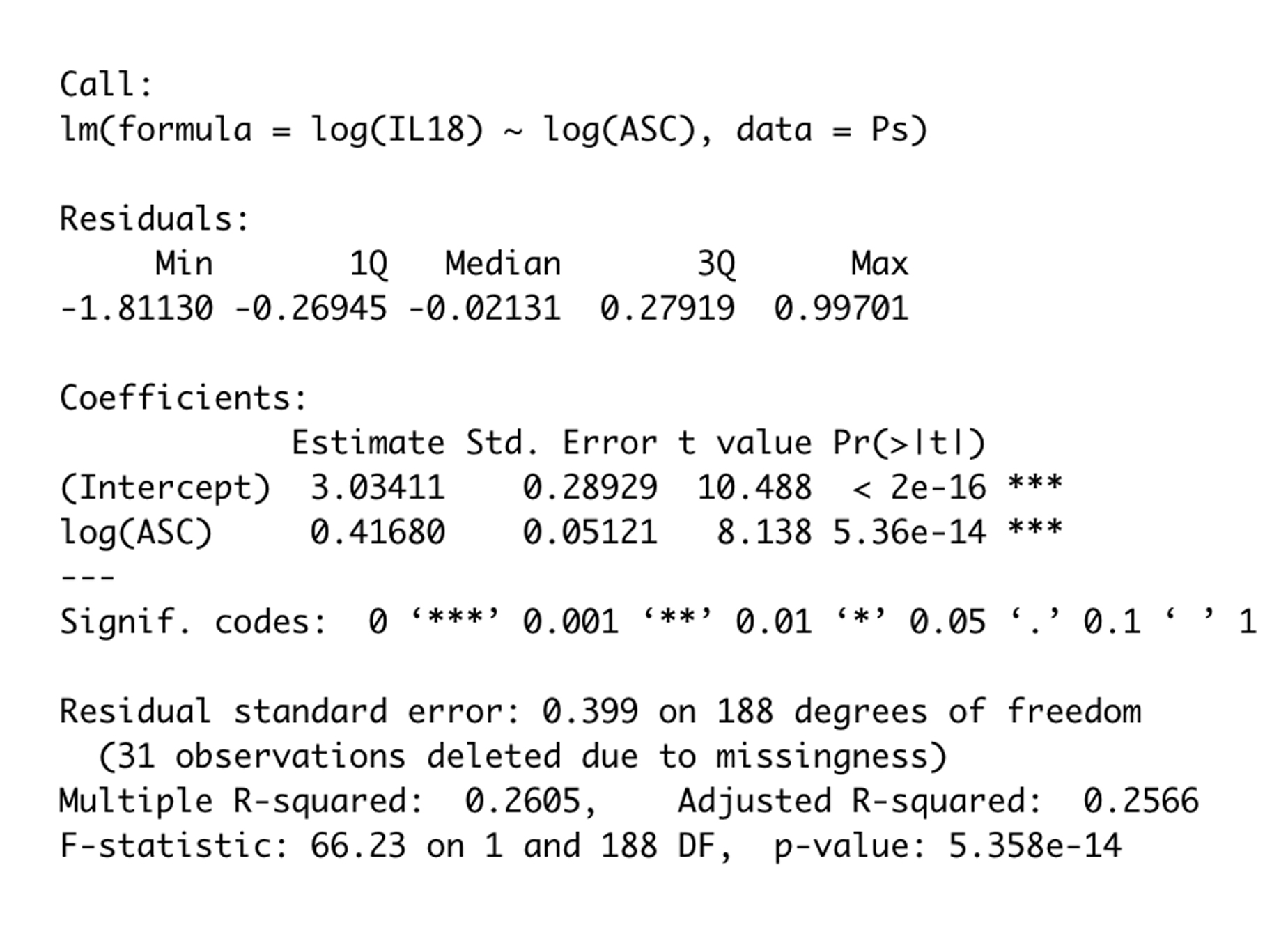

Supplement: Supplementary Figure 1 — Linear regression model fit results for Log(IL-18) ~ Log(ASC). [file Image_1.jpeg]

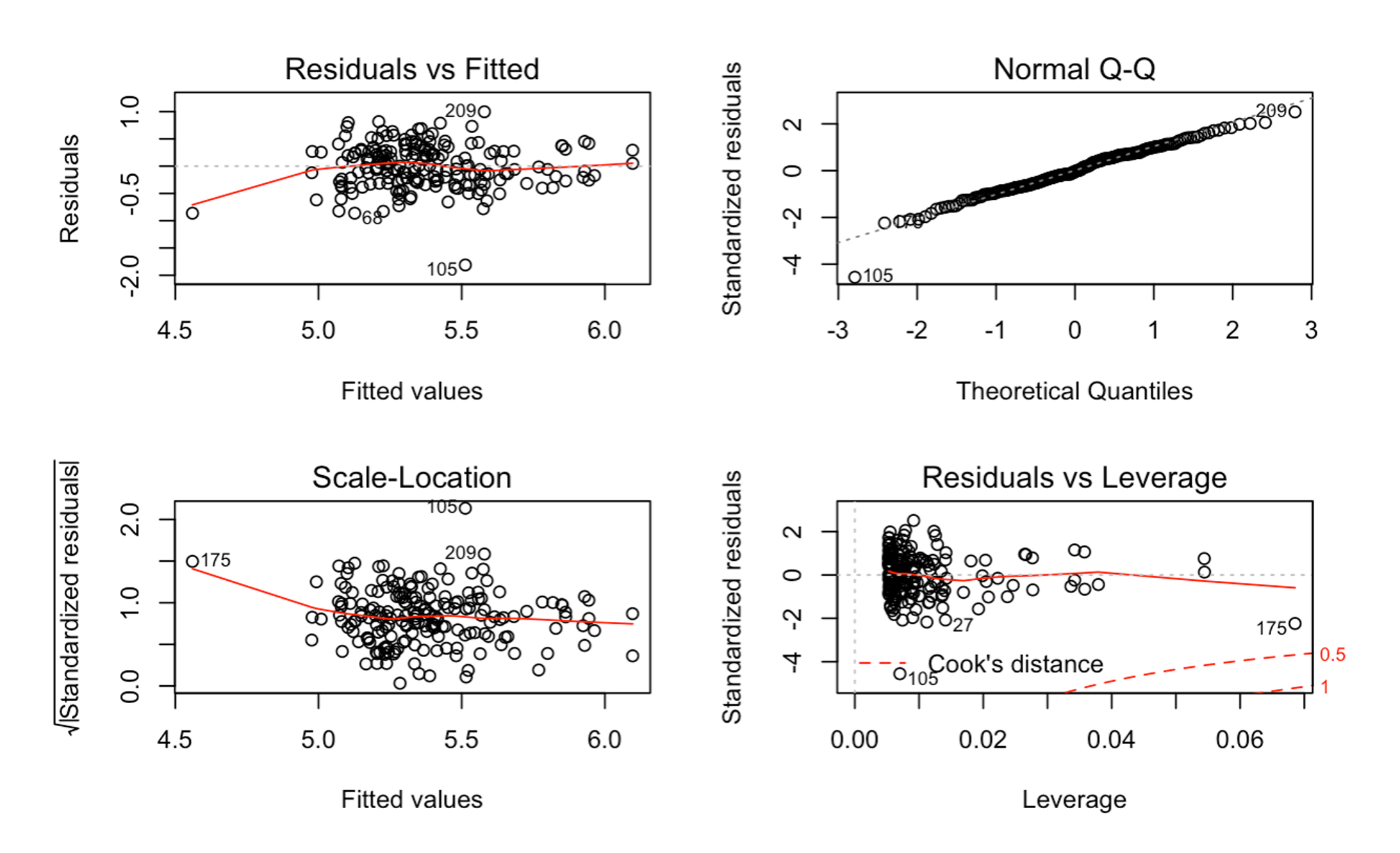

Supplement: Supplementary Figure 2 — Residual analysis results for the model fit for Log(IL-18) ~ Log(ASC). [file Image_2.jpeg]

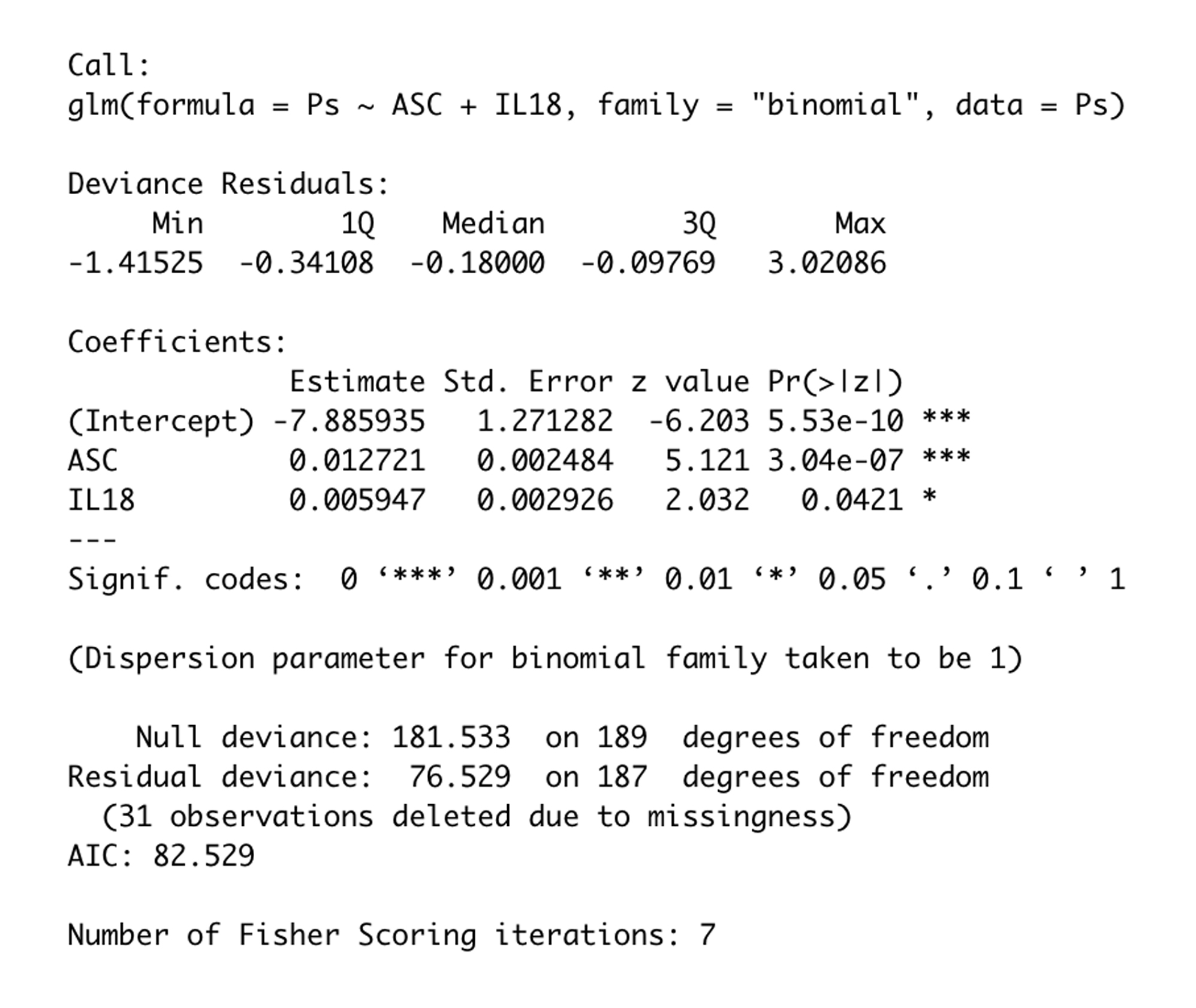

Supplement: Supplementary Figure 3 — Logistic regression model fit results for Ps ~ ASC + IL-18. [file Image_3.jpeg]
